# Prevalence and Risk Factors of Gallbladder Polyps Among Jeju Natives and Migrants: Retrospective Cross-Sectional Study

**DOI:** 10.3390/jcm15103863

**Published:** 2026-05-17

**Authors:** Oh-Sung Kwon, Young-Kyu Kim

**Affiliations:** 1 Department of Medical Information, Jeju National University Hospital, Jeju-si 63241, Jeju Special Self-Governing Province, Republic of Korea; dhtjd2@daum.net; 2 Department of Surgery, Jeju National University Hospital, Jeju-si 63241, Jeju Special Self-Governing Province, Republic of Korea

**Keywords:** gallbladder polyps, Jeju Island, natives vs. migrants, HDL-cholesterol, alcohol consumption, ultrasonography

## Abstract

**Background/Aim**: Routine ultrasonography now detects gallbladder polyps (GBPs) more often, but reported prevalence varies across populations. Jeju Island has South Korea’s highest obesity prevalence and red meat consumption, suggesting a rapid nutritional transition similar to that reported in Native Hawaiians. This study aimed to first analyze risk factors influencing GBP prevalence, including birthplace, and second compare clinical variables between JNs and JMs. **Methods**: Between May 2018 and October 2023, 28,751 individuals underwent medical checkups at Jeju National University Hospital. GBPs were diagnosed via ultrasonography, and risk factors including age, sex, birthplace, metabolic syndrome, hepatitis B virus antigen (HBsAg) positivity, lipid profiles, and alcohol consumption were assessed using univariate and multivariate logistic regression. **Results**: After exclusions, 15,219 participants were analyzed. The overall prevalence of GBPs was 10.3%. Male participants had a higher prevalence than females (11.4% vs. 9.1%, *p* < 0.001). The younger age group (20–49 years) showed the highest prevalence, while those aged ≥70 years had the lowest (11.6% vs. 8.6%, *p* = 0.001). Jeju Natives (JNs) exhibited a significantly higher prevalence than Jeju migrants (JMs) (10.6% vs. 9.0%, *p* = 0.004). Multivariate analysis identified female sex [odds ratio (OR) = 0.644, *p* < 0.001], age ≥ 70 years (OR = 0.601, *p* < 0.001), JN birthplace (OR = 1.260, *p* = 0.015), HBsAg positivity (OR = 1.347, *p* = 0.040), and high-risk alcohol drinking (OR = 0.758, *p* = 0.001) as independent predictors. Notably, the 60–69 age group did not reach statistical significance in the optimized model (*p* = 0.158). Compared to JMs, JNs were older and had a higher prevalence of fatty liver disease, a higher BMI, and higher levels of AST and GGT, but lower levels of HDL-cholesterol and triglycerides. **Conclusions**: GBPs are more prevalent among JNs compared to JMs, with birthplace emerging as a novel independent risk factor. Fatty liver disease, BMI, and reduced HDL-cholesterol were associated with GBP risk. These findings hypothesize that dietary and metabolic health factors may be potential pathways for the higher GBP prevalence among JNs, though direct dietary assessment is required for confirmation.

## 1. Introduction

Gallbladder polyps (GBPs), defined as polypoid lesions protruding from the mucosa into the lumen of the gallbladder, are frequently detected by ultrasonography [1]. The prevalence of GBPs has been reported as 4–7% in Western countries [2], 6–9% in Asia [2], and approximately 9–10% in Far East Asia [3]. With the increasing use of ultrasound in clinical practice, GBPs are often discovered incidentally during health screenings [4].

Clinical guidelines recommend periodic ultrasonographic surveillance for patients with GBPs ≥5 mm who lack risk factors for gallbladder cancer [5]. GBPs pose a clinical dilemma because non-invasive imaging often fails to differentiate rare malignant or precancerous neoplastic polyps from benign pseudopolyps. Although neoplastic polyps can progress to adenocarcinoma through the adenoma–carcinoma sequence, lesions smaller than 5 mm are typically non-neoplastic, whereas those ≥10 mm carry a 3–8% risk of malignancy [4,5]. For GBPs ≥10 mm or in the presence of established malignancy risk factors, guidelines strongly recommend laparoscopic cholecystectomy [6]. Incidental GBPs can increase downstream testing and treatment, increasing healthcare expenditures.

Known risk factors for GBPs include male sex, middle age, metabolic syndrome (MS), hepatitis B virus antigen (HBsAg) positivity, dyslipidemia, and dietary habits—particularly high intake of red meat and high-fat diets [7]. Among these, diet is a modifiable risk factor, and dietary changes can reduce GBP incidence, thereby lowering healthcare costs [8].

Jeju Island, located approximately 80 km from the mainland, consistently reports the highest obesity and red meat consumption rates in South Korea [9]. According to the Korea National Health and Nutrition Examination Survey-based Follow-up Survey (KNHANES) (2018–2023), Jeju residents consume more fat and red meat than those in other provinces and have the highest obesity prevalence nationwide [10].

Similar trends have been observed among Native Hawaiians in the United States, whose obesity prevalence is approximately 1.5-fold higher than that of Asians or non-Hispanic Whites living on the same islands [11]. This disparity may be associated with a nutritional transition from traditional seafood-based diets to lower-cost, energy-dense Westernized foods, including fast food, processed foods, and sugar-sweetened beverages.

The expansion of low-cost carriers and ferry services since 2008 has led to population growth and the proliferation of fast-food franchises. Concurrently, since 2014, the number of Jeju migrants (JMs) has increased significantly, accounting for approximately 30% of the population by 2023 [12]. KNHANES 2023 reported that Jeju residents lead the nation in red meat and salt intake and have maintained the highest obesity rate since 2018 [13].

We hypothesized that Jeju Natives (JNs), similar to Native Hawaiians, may have undergone a rapid nutritional transition [11], which could be associated with obesity and impaired lipid metabolism due to high fat and red meat consumption [9,10,13]. Consequently, JNs may exhibit a higher prevalence of GBPs compared to JMs, potentially related to nutritional and metabolic factors, although these remain speculative. This study aimed to first analyze risk factors influencing GBP prevalence, including birthplace, and second compare clinical variables between JNs and JMs.

## 2. Materials and Methods

### 2.1. Participants

**Figure 1:**
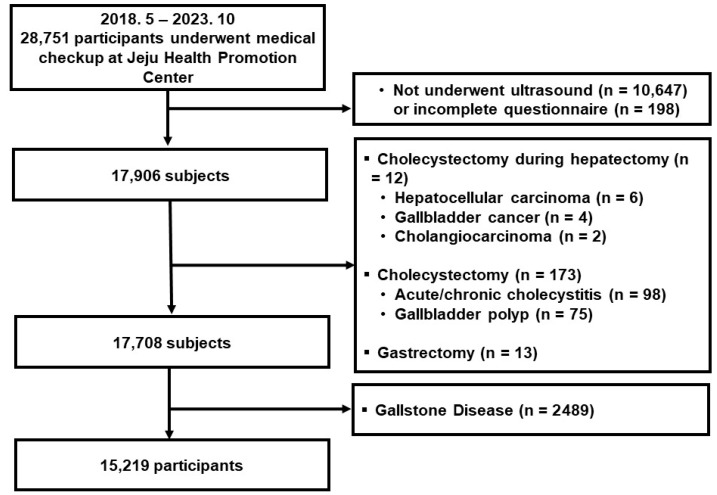
Flow diagram of included participants who underwent medical checkups.

The inclusion criteria for this study were adults aged 20 years or older who had undergone a medical examination at the hospital; the exclusion criteria were (1) those who did not undergo abdominal ultrasonography, (2) those who did not respond to the survey or who provided incomplete answers, (3) those whose gallbladder had been removed due to previous hepatobiliary surgery or who had undergone gastrectomy resulting in gallbladder dyskinesia, and (4) those with gallbladder stones. Between May 2018 and October 2023, a total of 28,751 individuals underwent medical examinations at the Health Screening and Promotion Center of Jeju National University Hospital. Of these, 10,647 who did not undergo ultrasonography and 198 with incomplete questionnaires were excluded, leaving 17,906 eligible participants. Subsequently, individuals with a history of cholecystectomy during hepatectomy (*n* = 12), hepatocellular carcinoma (*n* = 6), gallbladder cancer (*n* = 4), cholangiocarcinoma (*n* = 2), prior cholecystectomy (*n* = 173), acute or chronic cholecystitis (*n* = 98), or previously diagnosed GBPs (*n* = 75) were excluded, resulting in 17,708 subjects. Thereafter, participants with a history of gastrectomy (*n* = 13) and those diagnosed with gallstone disease (*n* = 2489) were further excluded, resulting in the inclusion of 15,219 participants in the final analysis (Figure 1). The study was conducted in accordance with the ethical standards of the Institutional Review Board (IRB), which granted a waiver of informed consent as the research posed minimal risk to participants. The study was approved by the IRB of Jeju National University Hospital (IRB number: 2024-06-003).

### 2.2. Questionnaire

**Figure 2:**
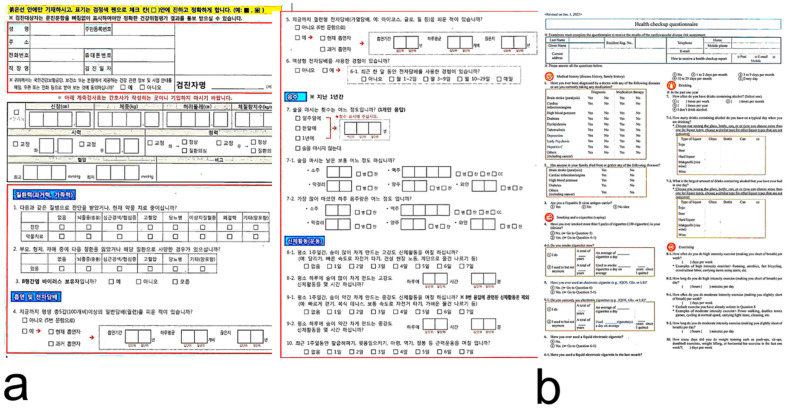
The questionnaire used in this study followed the format of the 2018 Korea National Health and Nutrition Examination Survey. Since Korean is the official language of South Korea, the survey was administered in Korean. The original Korean version (**a**) and the English translation (**b**).

Subjects completed a demographic and clinical questionnaire that included the following items: birthplace, telephone number, address, medical history (including diabetes mellitus, hyperlipidemia, hypertension, stroke, heart disease, tuberculosis, and related medication history), other medications, and alcohol consumption. We minimized self-report bias by using objective and graded questions. The questionnaire used in this study followed the format of the 2018 KNHANES. Since Korean is the official language of South Korea, the survey was administered in Korean. To ensure the transparency of the research instruments, the original Korean version (Figure 2a) and the English translation (Figure 2b) are provided. Participants were blinded to the study objectives while completing the questionnaire to reduce recall bias. Birthplace was identified using Resident Registration Numbers in medical records. Subjects were divided into JNs, defined as individuals with Resident Registration Numbers identifying Jeju, and JMs, defined as individuals with other registration numbers.

### 2.3. Diagnosis of GBPs and Fatty Liver Disease via Ultrasound

Ultrasound examinations were performed by specialized radiologists using high-resolution ultrasound equipment (Koninklijke Philips Electronics N.V., Amsterdam, The Netherlands) [14]. Subjects underwent abdominal ultrasound scans after fasting for more than 8 h. Radiologists identified GBPs as hyperechoic, fixed lesions protruding from the gallbladder mucosa into the lumen without acoustic shadow, which remained stationary despite changes in the subject’s position [15]. For each subject, the size of the largest polypoid lesion and the number of polyps were recorded. Fatty liver disease was diagnosed by expert radiologists based on fine parenchymal echotexture, blurring of intrahepatic vessels, increased hepatic echogenicity compared with the kidney, poor beam penetration, and poor diaphragmatic echo contrast [16]. Participants with gallstone disease were excluded to ensure diagnostic accuracy, as acoustic shadowing can interfere with the identification of small gallbladder polyps. Because this study was retrospective in nature, the radiologists who performed the abdominal ultrasonography were completely unaware of the study objectives at the time of examination. Therefore, their assessments were not influenced by participants’ birthplace or clinical data, and the imaging results were not biased by knowledge of the research.

### 2.4. Definition of Physical Activity, Alcohol Consumption, and MS

Physical activity levels were assessed according to the WHO Global Recommendations on Physical Activity for Health (2010) [17]. Participants were defined as physically active if they did vigorous-intensity physical activity for ≥75 min or moderate-intensity aerobic activity for ≥150 min, or an equivalent combination of moderate- and vigorous-intensity activity throughout the week, ensuring that each aerobic session lasts for at least 10 min. Alcohol consumption was calculated as pure alcohol intake (type × glasses × specific gravity of alcohol) and converted into Korean standard drinks (7 g of pure alcohol per glass) [18]. High-risk alcohol drinkers were defined as men who consumed seven or more drinks per occasion and women who consumed five or more drinks per occasion, with more than two drinking occasions per week [19]. MS was defined based on the revised National Cholesterol Education Program criteria [20]. Subjects were diagnosed with MS if they fulfilled three or more of the criteria.

### 2.5. Physical Examination

Height and weight were measured using an electronic combined scale and stadiometer (GL-150R, G-Tech International Co., Uijeongbu-si, Gyeonggi-do, Republic of Korea) with light clothing and without shoes. Age and sex were collected from medical records. Venous blood samples were obtained after 8 h of fasting. Laboratory tests included alanine aminotransferase (ALT), aspartate aminotransferase (AST), fasting blood glucose, alkaline phosphatase (ALP), gamma-glutamyl transferase (GGT), triglycerides, total cholesterol, low-density lipoprotein (LDL)-cholesterol, and high-density lipoprotein (HDL)-cholesterol.

GBP prevalence was calculated according to age and sex. Participants were divided into four age groups: 20–49, 50–59, 60–69, and ≥70 years. Body mass index (BMI) was calculated as weight divided by height squared (kg/m^2^) and categorized according to WHO criteria for Asian populations: <18.5 (underweight), 18.5–22.9 (normal weight), 23.0–24.9 (overweight), and ≥25.0 (obese). Fasting blood glucose was classified into three groups based on ADA 2015 standards: <100 mg/dL (normoglycemia), 100–125 mg/dL (impaired fasting glucose), and ≥126 mg/dL (diabetes mellitus). Lipid profiles were categorized according to the Korean Guidelines for the Management of Dyslipidemia (2023, 5th edition) [21]. Elevated AST was defined as >32 IU/L for men and >26 IU/L for women. Elevated ALT was defined as >34 IU/L for men and >24 IU/L for women [22]. Elevated ALP and GGT were defined as >71 IU/L and >130 IU/L, respectively.

### 2.6. Statistical Analysis

Univariate binary logistic regression analysis was performed to evaluate potential independent risk factors for GBPs, including age, sex, birthplace, AST, ALT, BMI, fasting glucose, triglycerides, total cholesterol, HDL-cholesterol, LDL-cholesterol, and ALP. Multivariable logistic regression analysis was performed to identify independent predictors of GBPs. Candidate variables were initially screened via univariate analysis, and those with a *p*-value <0.1 were entered into the multivariable model. The final model was constructed using a backward stepwise (Wald) elimination process. This method was employed to optimize the statistical power of the model and ensure mathematical consistency between the odds ratios and their corresponding confidence intervals. Results are presented as adjusted odds ratios with 95% confidence intervals. Clinical variables were compared using the chi-square test for categorical variables and Student’s *t*-test for continuous variables according to birthplace (JNs vs. JMs). Because some laboratory variables contained missing values, denominators varied across variables. For descriptive statistics, percentages were calculated using the available data for each variable. Regression analyses were conducted using complete-case analysis, whereby participants with missing data for any covariates in the model were excluded. The extent of missingness for each variable is reported in Table 1. Statistical significance was set at *p*-value < 0.05. All statistical analyses were performed using PASW Statistics for Windows, version 18.0 (SPSS Inc., Chicago, IL, USA).

## 3. Results

### 3.1. Sex and Annual Prevalence of GBPs

Among the 15,219 participants, 7774 were men (51.1%) and 7445 were women (48.9%). The annual prevalence of GBPs was recorded as 12.0% in 2018 (May–December), 11.2% in 2019, 8.7% in 2020, 9.3% in 2021, 8.5% in 2022, and 11.7% in 2023 (up to October). There was no statistical correlation between annual GBP prevalence and study years.

### 3.2. Univariate Analysis of Risk Factors for GBPs

Factors associated with the presence of GBPs are summarized in Table 1. The prevalence of GBPs was 11.6% in the 20–49-year age group, 10.4% in the 50–59-year group, 10.2% in the 60–69-year age group, and 8.6% in the ≥70-year age group (*p* = 0.001). Male sex, JNs, MS, BMI, HDL-cholesterol level, GGT level and AST level, HBsAg positivity and high-risk alcohol drinker were significant risk factors for GBPs.

### 3.3. Multivariate Analysis of Risk Factors for GBPs

Binary logistic regression analyses were performed for clinical variables, including Male sex, age, JNs, MS, BMI, fasting blood glucose level, HDL-cholesterol level, GGT level, AST level, HBsAg positivity and high-risk alcohol drinker, among participants with GBPs. In the optimized multivariable model, female sex, JN birthplace, HBsAg positivity, and non-high-risk alcohol drinking were identified as independent predictors (Table 2). While the 20–49 age group showed a significant association, the 60–69 age group did not independently reach statistical significance (odd ratio = 0.880, 95% CI 0.737–1.051, *p* = 0.158).

### 3.4. Comparison of Clinical Variables Between JNs and JMs

Participants were divided into JNs and JMs according to their birthplace to investigate intergroup differences (Table 3). JNs were significantly older than JMs (*p* < 0.001). They showed significantly higher levels of AST (*p* = 0.038) and GGT (*p* = 0.007), but lower levels of HDL-cholesterol (*p* < 0.001) and triglycerides (*p* = 0.003). Additionally, JNs had a higher prevalence of fatty liver disease (*p* = 0.026) and a higher BMI (*p* < 0.001).

## 4. Discussion

The prevalence of GBPs in this study was 10.3%, which is higher than that reported in studies conducted among Asian groups, Far East Asian groups, and other South Korean populations [23]. One possible explanation for this discrepancy is the continuous improvement in the resolution of abdominal ultrasound equipment [24], which allows for the detection of smaller lesions that may have been missed in earlier studies. However, since our institution has used the same high-resolution ultrasound equipment since 2017, the detection rate of GBP lesions should not have varied across the study period. Therefore, it is plausible that the actual prevalence of GBPs has increased in the Jeju population.

Jeju residents have the highest obesity rate in South Korea [25]. In a comparative context, Native Hawaiians have undergone a pronounced nutritional transition, shifting from traditional seafood-based diets to processed, high-calorie, and high-fat Western foods. This transition has been closely associated with rising obesity and metabolic disease prevalence in these communities. At the community level, nutritional transitions and socioeconomic drivers can elevate the risk of gallbladder disease by altering dietary patterns, reducing physical activity, and worsening metabolic health. Recent community-engaged studies and public health initiatives among Native Hawaiians and other Pacific Islander groups emphasize that tackling obesity and diet-related health inequities requires context-specific, community-based interventions and policies that improve food access, strengthen nutrition education, and address structural determinants of health. These parallels support the use of Native Hawaiian population trends as a contextual example of how rapid dietary Westernization at the population level may contribute to increased gallbladder pathology.

Similarly, JNs have shifted from traditional seafood diets to Westernized diets dominated by processed red meat and high-fat foods [25,26]. This dietary change is a plausible factor that correlates with the higher GBP prevalence, suggesting a need for future studies with direct dietary data. Consistent with this interpretation, KNHANES 2023 reported that lipid-derived energy intake increased by more than 5% in both men and women over the past decade [27].

Age was also found to be an important risk factor [1,2,3,7,8,9]. Although different studies have reported varying age thresholds, with some identifying middle age as under 60 years and others as over 60 years, our study found that belonging to the 20–49-age group was an independent risk factor for GBP prevalence. In the multivariable analysis, although the 60–69 age group was initially considered, we further refined our model using a backward stepwise selection process to optimize the statistical power, as suggested during the peer-review process. This optimized analysis confirmed that the 20–49 age group remains a robust independent risk factor, whereas the association for the 60–69 group did not reach statistical significance (*p* = 0.158). This adjustment ensures the mathematical consistency and reliability of our findings, highlighting that the impact of age is most pronounced in the younger population within our study cohort. According to KNHANES 2023, individuals’ energy intake increases in their 40s and peaks in their 50s before declining. This decline in older age may be explained by increased prevalence of periodontal disease and reduced digestive capacity, which lead to decreased food intake. Consequently, lipid profiles improve, reducing the risk of GBP development.

The most notable finding of this study is that JN birthplace emerged as an independent risk factor for GBP prevalence in multivariate analysis. To explore this further, we compared JNs and JMs. JNs exhibited a higher prevalence of fatty liver disease, elevated levels of GGT and AST, and lower HDL-cholesterol levels. HDL-cholesterol plays a crucial role in reverse cholesterol transport, removing excess cholesterol from the gallbladder wall and preventing the formation of cholesterol polyps [28]. This metabolic vulnerability is particularly concerning given that Jeju residents consistently report higher obesity rates and higher fat/red meat consumption compared to those in other provinces, as documented in national health surveys [10,13,25]. Therefore, the relatively impaired lipid profile and metabolic risk observed in JNs likely contribute to their higher prevalence of GBPs, reinforcing the need for targeted metabolic interventions and dietary education in this group. Our multivariate analysis revealed that high-risk alcohol consumption was independently associated with lower odds of GBPs (odds ratio = 0.758, *p* < 0.001). This finding aligns with several previous studies and animal experiments suggesting that alcohol consumption may exert a protective effect against GBP formation [29,30]. The potential mechanism involves alcohol-induced stimulation of cholecystokinin secretion, which enhances gallbladder contraction and subsequently reduces cholesterol saturation in the bile [29]. While JNs exhibited higher levels of AST and GGT, which often reflect hepatic stress or alcohol intake, our data suggest that the physiological impact of alcohol on gallbladder motility might paradoxically reduce the incidence of polypoid lesions in this population. In other words, alcohol consumption may paradoxically inhibit, rather than promote [31] the development of GBPs through its stimulatory effect on gallbladder motility, potentially depending on specific consumption thresholds. Our finding that high-risk alcohol drinking was independently associated with lower odds of GBPs is consistent with some previous studies; however, this must be viewed as a highly speculative hypothesis requiring cautious interpretation. While some suggest that alcohol might affect bile cholesterol saturation, the potential ‘protective’ effect observed in this cross-sectional study is likely paradoxical. Given the established detrimental effects of high-risk drinking on overall metabolic health and its role as a known carcinogen, this association should not be interpreted as a clinical recommendation. Further longitudinal studies are necessary to clarify the biological mechanisms underlying this speculative association.

This study revealed that JNs have a significantly higher prevalence of GBPs compared to JMs, despite paradoxically lower serum TG levels. Two mechanisms may explain this finding. First, the lower TG levels in JNs may reflect a lipid sequestration phenomenon, whereby lipids are preferentially deposited into tissues such as the liver and gallbladder wall rather than circulating in blood. This interpretation is supported by the higher BMI and greater prevalence of fatty liver disease observed in JNs, suggesting a metabolic predisposition toward tissue lipid accumulation despite reduced serum concentrations [32]. Second, reduced HDL-cholesterol in JNs likely plays a more decisive role in GBP pathogenesis. HDL-cholesterol is essential for reverse cholesterol transport, which clears excess cholesterol from peripheral tissues including the gallbladder mucosa. Impaired clearance due to low HDL-cholesterol promotes foam cell formation and accelerates cholesterol polyp development [28,33]. GBP formation in JNs is potentially associated with a metabolic profile of lipid sequestration and impaired cholesterol clearance, representing a speculative mechanism based on our cross-sectional data. This metabolic profile may reflect the impact of a rapid nutritional transition unique to the JN population, offering a plausible explanation for the paradox of lower serum TG alongside a higher burden of gallbladder disease.

Laparoscopic cholecystectomy is established as the current gold standard for managing GBPs that are ≥1 cm with low suspicion of malignancy, offering favorable perioperative outcomes and reduced morbidity. However, the paramount technical priority during laparoscopy remains the prevention of iatrogenic gallbladder perforation: in cases where a polyp possesses malignant potential, intraoperative rupture significantly increases the risk of peritoneal seeding and subsequent carcinomatosis, which carries a dismal prognosis [1,4,5]. Consequently, a low threshold for conversion to open laparotomy is advised when dense adhesions or severe chronic inflammation may compromise the integrity of the gallbladder wall. Although novel techniques such as endo-laparoscopic polypectomy have been explored to preserve the gallbladder, they are currently not recommended in clinical practice owing to the inherent risk of transmural dissemination.

The clinical challenge of overdiagnosis in GBP management is further underscored by the POLYP study [34]. This prospective multicenter study demonstrated that the revised 2022 European guidelines only marginally improved surgical specificity compared with the 2017 version, with the indication for cholecystectomy remaining valid for approximately 77–81% of operated patients under both frameworks. However, histopathological outcomes revealed a significant gap between guideline indications and clinical reality: only 6 of 68 patients (9%) with a valid surgical indication actually harbored an adenoma. The authors argue that heavy reliance on a 10 mm size cutoff and on growth patterns in 6–9 mm polyps may lead to unnecessary morbidity and healthcare costs. Consequently, they advocate a shift toward a multifactorial approach, with a shortened follow-up duration of 2 years and the integration of advanced diagnostic tools, such as contrast-enhanced ultrasound, to better identify true neoplastic potential.

In light of the high rate of unwarranted cholecystectomies reported in the POLYP study [34], UDCA (Ursodiol) warrants consideration as a potential first-line conservative strategy for incidental GBPs. Because many resected cases are pathologically confirmed as non-neoplastic pseudopolyps, a 3- to 6-month trial of UDCA can serve as a valuable diagnostic-therapeutic tool. By reducing biliary cholesterol saturation, it promotes the dissolution of cholesterol polyps, which are often indistinguishable from true adenomas on standard ultrasonography. In a significant subset of patients with small (<10 mm), asymptomatic lesions, this approach could eliminate the need for long-term surveillance or invasive surgery. However, it is crucial to note that UDCA is ineffective against neoplastic tissue; therefore, its use must be strictly limited to low-risk cases and should not delay surgical intervention if high-risk features, such as rapid growth or size ≥ 10 mm, are present.

Abdominal ultrasonography remains the primary non-invasive modality for detecting gallbladder polyps (GBPs), which are often discovered incidentally. A recent multicenter study in Qatar [35] involving 7156 individuals reported an overall GBP prevalence of 7.4%. In that study, most detected lesions were small, with 89.4% measuring <6 mm and only 1.3% reaching the high-risk threshold of ≥10 mm. The prevalence of GBPs in our study (10.3%) is notably higher than the 7.4% reported in the Qatar study. Specifically, the prevalence of GBPs was 1.5% for lesions ≥ 10 mm, 20.2% for those between 6 and 9 mm, and 78.3% for those <6 mm. While the proportion of participants meeting the indications for laparoscopic cholecystectomy was comparable to that of the Qatar study, there was a higher percentage of patients requiring pharmacological intervention and close ultrasonic surveillance.

Consequently, for regions where a Westernized high-fat diet contributes to a high prevalence of GBPs, we propose a tailored clinical strategy for the majority of patients who are not candidates for immediate surgery. This approach combines a trial of Ursodiol therapy (particularly for lesions < 6 mm) with structured radiological surveillance (every 6–12 months for lesions ≥ 6 mm). Such a strategy offers an effective non-invasive management framework, ultimately reducing the burden of unnecessary, prolonged radiological monitoring.

This study has six primary limitations. First, Birthplace was determined using social registration numbers; therefore, we could not accurately assess how long individuals had resided on Jeju Island or how dietary habits changed with longer residence. Therefore, the proposed link between a Westernized diet and higher GBP prevalence among JNs should be interpreted as a preliminary hypothesis rather than a causal conclusion, and it warrants further validation through prospective research. Future studies should examine duration of residence and evaluate how it affects GBP prevalence through dietary change. Second, this cross-sectional observational study was conducted at a single health screening center at Jeju National University Hospital, which limits generalizability. Future multicenter cohort observational studies are necessary to validate these results. Third, although ultrasonography is effective for detecting GBPs, it cannot definitively distinguish among histological types. As a result, we were unable to classify polyps by type, which could have clarified their clinical significance. Fourth, the severity of fatty liver disease was assessed using ultrasound criteria. Although ultrasound is widely used, it may be less accurate than newer technologies such as vibration-controlled transient elastography, which has been shown to provide higher diagnostic accuracy in non-alcoholic fatty liver disease [36,37]. This limitation may have reduced the precision of fatty liver diagnosis in our study. Fifth, birthplace was defined by Resident Registration Numbers, which serve as reliable administrative records but do not capture total residence duration or migration timing. This potential misclassification may have diluted observed associations. Future studies incorporating detailed residential histories are needed. Lastly, this study did not directly measure cortisol levels or medication history, both of which may play a role in the pathophysiology of gallbladder and hepatic diseases, because our research was primarily designed to focus on community-based epidemiology and lifestyle factors. Future studies incorporating these endocrine biomarkers would provide a more comprehensive understanding of the disease mechanisms. Despite these limitations, this is the first study to divide Jeju residents into two groups based on birthplace—JNs and JMs—and to report that GBP prevalence is significantly higher among JNs within a genetically similar population.

## 5. Conclusions

This study identified a 10.3% prevalence of GBPs in the Jeju population, with JNs exhibiting a significantly higher prevalence than JMs. Multivariate analysis demonstrated that specific age groups (20–49 years), male sex, JN birthplace, HBsAg positivity, and non-high-risk alcohol drinking were independent risk factors for GBPs. Notably, age- and sex-related differences were also evident. Additionally, the elevated liver enzymes and higher prevalence of fatty liver disease observed among JNs may reflect distinct patterns of alcohol consumption and an underlying metabolic vulnerability, both of which are likely associated with the rapid nutritional transition of the Jeju-born population.

This study showed that individuals born on Jeju Island are significantly more likely to have GBPs. These findings suggest that dietary and metabolic health factors may be potential pathways requiring further prospective investigation that address the distinct dietary and lifestyle transitions in the JN population. Deteriorating metabolic health, potentially driven by these transitions, shows a significant association with the burden of gallbladder disease on Jeju Island. Public health strategies are warranted for populations undergoing rapid nutritional change, including targeted dietary and lifestyle interventions, strengthened nutrition education, improved access to healthy foods, and focused management of MS and liver disease. Future research should incorporate JM residence duration, the precise quantification of alcohol intake, the histological characterization of polyps, and multicenter prospective cohort studies to better elucidate causal mechanisms.

## Figures and Tables

**Table 1:** Results of univariate analysis of risk factors for GBPs.

GBPs					
Factors	Total Participants, *n*	GBPs, *n* (%)	OR (95% CI)	* *p* Value	Missing
Sex				<0.001	0
Male	7774	885 (11.4)	1.000		
Female	7445	675 (9.1)	0.776 (0.698–0.863)		
Age (years)				0.001	0
20–49	3318	385 (11.6)	1.000		
50–59	4259	442 (10.4)	0.882 (0.763–1.020)	0.090	
60–69	4697	481 (10.2)	0.869 (0.754–1.002)	0.053	
≥70	2945	252 (8.6)	0.713 (0.603–0.843)	<0.001	
Birthplace				0.004	0
JN	11,644	1239 (10.6)	1.207 (1.061–1.373)		
JM	3575	321 (9.0)	1.000		
Fatty liver disease				0.121	0
Yes	8235	873 (10.6)	1.087 (0.978–1.208)		
No	6984	687 (9.8)	1.000		
Metabolic syndrome				0.003	0
Yes	4380	469 (10.7)	1.197 (1.062–1.349)		
No	10,839	986 (9.1)	1.000		
BMI (kg/m^2^)				0.008	14
<18.5	243	19 (7.8)	1.000		
18.5–22.9	4220	384 (9.1)	1.180 (0.730–1.907)	0.499	
23–24.9	3811	427 (11.2)	1.488 (0.921–2.402)	0.104	
≥25	6931	728 (10.5)	1.384 (0.861–2.224)	0.180	
Fasting blood glucose (mg/dL)				0.064	9
<100	9755	839 (8.6)	1.000		
100–125	4027	403 (10.0)	1.255 (1.032–1.527)	0.023	
≥126	1428	151 (10.6)	1.183 (0.958–1.462)	0.119	
Total cholesterol (mg/dL)				0.328	3154
<200	6376	661 (10.4)	1.000		
200–239	3873	380 (9.8)	0.941 (0.823–1.074)	0.367	
≥240	1816	168 (9.3)	0.881 (0.738–1.053)	0.164	
LDL-cholesterol (mg/dL)				0.499	3357
<100	6215	628 (10.1)	1.000		
100–129	3514	354 (10.1)	0.997 (0.869–1.143)	0.962	
130–159	1631	172 (10.5)	1.049 (0.878–1.253)	0.600	
160–189	408	36 (8.8)	0.861 (0.606–1.224)	0.404	
≥190	94	5 (5.3)	0.500 (0.202–1.235)	0.133	
HDL-cholesterol (mg/dL)				0.017	3139
<40	1077	123 (11.4)	1.000		
40–60	5780	608 (10.5)	0.912 (0.742–1.120)	0.379	
≥60	5223	479 (9.2)	0.783 (0.635–0.966)	0.022	
Triglycerides (mg/dL)				0.408	3132
<150	9447	968 (10.2)	1.000		
150–199	996	93 (9.3)	0.902 (0.721–1.128)	0.367	
200–499	1252	117 (9.3)	0.903 (0.738–1.105)	0.321	
≥500	92	6 (6.5)	0.611 (0.266–1.402)	0.245	
AST (IU/L)				0.012	4
≤32 for men, ≤26 for women	12,226	1100 (9.0)	1.000		
>32 for men, >26 for women	2989	317 (10.6)	1.193 (1.039–1.369)		
ALT (IU/L)				0.399	3195
≤34 for men, ≤24 for women	11,824	1221 (10.3)	1.000		
>34 for men, >24 for women	200	17 (8.5)	1.240 (0.752–2.045)		
GGT (IU/L)				0.047	22
≤71	13,204	1188 (9.0)	1.000		
>71	1993	207 (10.4)	1.180 (1.002–1.389)		
ALP (IU/L)				0.845	4453
≤130	828	79 (9.5)	1.000		
>130	9938	969 (9.8)	1.024 (0.805–1.304)		
Diabetes medication use				0.174	0
Yes	1402	129 (9.2)	0.877 (0.726–1.060)		
No	13,817	1431 (10.4)	1.000		
Dyslipidemia medication use				0.506	0
Yes	1666	163 (9.8)	0.944 (0.795–1.119)		
No	13,553	1397 (10.3)	1.000		
Hypertension medication use				0.368	0
Yes	3645	388 (10.6)	1.057 (0.936–1.194)		
No	11,574	1172 (10.1)	1.000		
HBsAg				0.005	7055
Yes	547	69 (12.6)	1.461 (1.121–1.903)		
No	7617	685 (9.0)	1.000		
Physical activity ^1^				0.498	0
Yes	2907	288 (9.9)	0.954 (0.834–1.092)		
No	12,312	1272 (10.3)	1.000		
High-risk alcohol drinker ^2^				0.023	0
Yes	5074	480 (9.5)	0.877 (0.783–0.982)		
No	10,145	1080 (10.6)	1.000		

GBP: gallbladder polyp; ALP: alkaline phosphatase; ALT: alanine aminotransferase; AST: aspartate aminotransferase; BMI: body mass index; CI: confidence interval; GGT: gamma-glutamyl transferase; HBsAg: hepatitis B virus antigen; HDL: high-density lipoprotein; JM: Jeju migrant; JN: Jeju native; LDL: low-density lipoprotein; OR: odds ratio. * Analyzed using binary logistic regression. Note: Denominators vary across variables due to missing laboratory values. The number of participants included for each variable and the extent of missingness are indicated in Table 1. Percentages are calculated based on available data (complete-case analysis). ^1^ Physical activity: ≥150 min/week of moderate-intensity aerobic activity or ≥75 min/week of vigorous-intensity aerobic activity, with aerobic activity lasting >10 min per session (WHO 2010).^2^ High-risk alcohol drinker: ≥7 drinks/occasion (men) or ≥5 drinks/occasion (women) and ≥2 times/week (1 drink = 7 g alcohol).

**Table 2:** Results of multivariable logistic regression analysis of risk factors for GBPs.

GBPs			
Factors	OR	95% CI	* *p* Value
Sex (%)			<0.001
Male	1.000		
Female	0.644	0.547–0.759	
Age (years)			<0.001
20–49	1.000		
50–59	0.799	0.658–0.971	0.024
60–69	0.880	0.737–1.051	0.158
≥70	0.601	0.459–0.787	<0.001
Birthplace			0.015
JN	1.260	1.046–1.517	
JM	1.000		
HBsAg			0.040
Yes	1.347	1.014–1.788	
No	1.000		
High-risk alcohol drinker			0.001
Yes	0.758	0.643–0.893	
No	1.000		

GBP: gallbladder polyp; CI: confidence interval; GBPs: gallbladder polyps; HBsAg: hepatitis B virus antigen. JM: Jeju migrant; JN: Jeju native; OR: odds ratio. * Analyzed using binary logistic regression.

**Table 3:** Between-group comparisons by birthplace (JNs vs. JMs).

Variables	JNs (*n* = 11,644)	JMs (*n* = 3575)	† *p* Value
Sex (%)			0.631
Male	6084 (52.3)	1885 (52.7)	
Female	5560 (47.7)	1690 (47.3)	
Fatty liver disease			0.026
Yes	6486 (55.7)	1916 (53.6)	
No	5158 (44.3)	1599 (46.4)	
Metabolic syndrome			0.476
Yes	3368 (28.9)	1012 (28.3)	
No	8276 (71.1)	2563 (71.7)	
Age (years)	59.2 ± 12.0	58.4 ± 11.9	<0.001
BMI (kg/m^2^)	25.0 ± 3.4	24.4 ± 3.5	<0.001
Fasting blood glucose (mg/dL)	100.3 ± 26.1	101.2 ± 27.3	0.069
Total cholesterol (mg/dL)	197.9 ± 40.8	199.5 ± 41.5	0.070
LDL-cholesterol (mg/dL)	99.9 ± 32.9	99.3 ± 33.6	0.421
HDL-cholesterol (mg/dL)	58.4 ± 15.9	60.2 ± 17.1	<0.001
Triglycerides (mg/dL)	115.8 ± 89.7	121.5 ± 87.7	0.003
AST (IU/L)	28.9 ± 43.2	27.4 ± 18.4	0.038
ALT (IU/L)	29.5 ± 53.3	28.4 ± 26.5	0.241
GGT (IU/L)	46.0 ± 84.0	41.5 ± 87.6	0.007
ALP (IU/L)	206.4 ± 73.8	203.9 ± 72.8	0.124
HBsAg			0.320
Yes	419 (6.9)	128 (6.2)	
No	5689 (93.1)	1928 (93.8)	
^1^ Physical activity			0.282
Yes	2202 (18.9)	705 (19.7)	
No	9442 (81.1)	2870 (80.3)	
^2^ High-risk alcohol drinker			0.874
Yes	3886 (33.4)	1188 (33.2)	
No	7758 (66.6)	2387 (66.8)	

ALP: alkaline phosphatase; ALT: alanine aminotransferase; AST: aspartate aminotransferase; BMI: body mass index; GGT: gamma-glutamyl transferase; HBsAg: hepatitis B virus antigen; HDL: high-density lipoprotein; LDL: low-density lipoprotein. Values are presented as *n* (%) or mean ± SD. *p* values were obtained using binary regression. † *p* values were obtained using the chi-square test. Note: Odds ratios were calculated using binary logistic regression. *p*-values for categorical variables were obtained using chi-square tests, and *p*-values for continuous variables were obtained using Student’s *t*-tests. The total number for each clinical variable may not equal the overall population (N = 15,219) due to missing laboratory values. ^1^ Physical activity: ≥150 min/week of moderate-intensity aerobic activity or ≥75 min/week of vigorous-intensity aerobic activity, with aerobic activity lasting >10 min per session (WHO 2010). ^2^ High-risk alcohol drinker: ≥7 drinks/occasion (men) or ≥5 drinks/occasion (women), ≥2 times/week (1 drink = 7 g alcohol).

## Data Availability

The data are not publicly available due to there being no appropriate site for uploading them at present. The data presented in this study are available upon request from the corresponding author.

## Abbreviations

The following abbreviations are used in this manuscript:

KNHANES	Korea National Health and Nutrition Examination Survey
GBP	gallbladder polyp
JN	Jeju native
JM	Jeju migrant
MS	Metabolic syndrome
GGT	gamma-glutamyl transferase
ALP	alkaline phosphatase
AST	aspartate aminotransferase
LDL	low-density lipoprotein
HDL	high-density lipoprotein
BMI	body mass index
